# Supplementary material for: From sequence to enzyme mechanism using multi-label machine learning
Source: BMC Bioinformatics. 2014 May 19;15:150. doi: 10.1186/1471-2105-15-150 (PMC4229970; doi:10.1186/1471-2105-15-150)
Supplement: Additional file 2 — Java code of ml2db. Additional file ml2db_code.tar.gz contains the Java source code to run the multi-label machine learning experiments and save the results to database. The code’s Javadoc is included. [file 1471-2105-15-150-S2.zip › additional file 2/ml2db/ml2db/doc/mulan/data/LabelsMetaData.html]

LabelsMetaData


JavaScript is disabled on your browser.


- Overview
- Package
- Class
- Use
- Tree
- Deprecated
- Index
- Help

- Prev Class
- Next Class

- Frames
- No Frames

- All Classes

- Summary:
- Nested |
- Field |
- Constr |
- Method

- Detail:
- Field |
- Constr |
- Method


mulan.data

## Interface LabelsMetaData

- All Known Implementing Classes:
  :   LabelsMetaDataImpl

  ---

    

  ```
  public interface LabelsMetaData
  ```

  Represents meta data about label attributes and their structure.
  The labels can be organized in hierarchical structure. If no hierarchy
  is defined between labels, they are stored in a flat structure.
  This means all labels are represented as root `LabelNode` element.

  Author:
  :   Jozef Vilcek

- - ### Method Summary

    Methods

    | Modifier and Type | Method and Description |
    | --- | --- |
    | `LabelsMetaData` | `clone()` Returns a deep copy of the `LabelsMetaDataImpl` instance. |
    | `boolean` | `containsLabel(java.lang.String labelName)` Determines if `LabelsMetaData` contains a label with specified name. |
    | `java.util.Set<java.lang.String>` | `getLabelNames()` Gets the names of all labels. |
    | `LabelNode` | `getLabelNode(java.lang.String labelName)` Gets the `LabelNode` specified by label name. |
    | `int` | `getNumLabels()` Gets the total number of `LabelNode` nodes. |
    | `java.util.Set<LabelNode>` | `getRootLabels()` Gets the unmodifiable `Set` of root `LabelNode` nodes of label attributes hierarchy. |
    | `boolean` | `isHierarchy()` Determines if there is a hierarchy defined between labels. |

- - ### Method Detail


    - #### getRootLabels

      ```
      java.util.Set<LabelNode> getRootLabels()
      ```

      Gets the unmodifiable `Set` of root `LabelNode`
      nodes of label attributes hierarchy.

      Returns:
      :   the `Set` of root nodes.


    - #### getLabelNode

      ```
      LabelNode getLabelNode(java.lang.String labelName)
      ```

      Gets the `LabelNode` specified by label name. The name is unique identifier
      of the node and corresponds to label attribute in the data set.
      If `LabelNode` for given label name does not exists, `null` is returned.

      Parameters:
      :   `labelName` - the name of label of which the node should be retrieved

      Returns:
      :   the `LabelNode` for specified label of `null`
          if `LabelNode` does not exists for specified label name


    - #### getLabelNames

      ```
      java.util.Set<java.lang.String> getLabelNames()
      ```

      Gets the names of all labels. The label name is a unique identifier among all
      labels in the meta data.

      Returns:
      :   the names of all labels.


    - #### containsLabel

      ```
      boolean containsLabel(java.lang.String labelName)
      ```

      Determines if `LabelsMetaData` contains a label with specified name.

      Parameters:
      :   `labelName` - the label name

      Returns:
      :   `true` if meta data contains the given label name;
          `false` otherwise.


    - #### isHierarchy

      ```
      boolean isHierarchy()
      ```

      Determines if there is a hierarchy defined between labels. If not, all labels are
      represented as root `LabelNode` nodes.

      Returns:
      :   `true` if there is hierarchy defined between labels;
          `false` otherwise.


    - #### getNumLabels

      ```
      int getNumLabels()
      ```

      Gets the total number of `LabelNode` nodes.

      Returns:
      :   total number of `LabelNode` nodes


    - #### clone

      ```
      LabelsMetaData clone()
      ```

      Returns a deep copy of the `LabelsMetaDataImpl` instance.

      Returns:
      :   a deep copy of the `LabelsMetaDataImpl` instance


- Overview
- Package
- Class
- Use
- Tree
- Deprecated
- Index
- Help

- Prev Class
- Next Class

- Frames
- No Frames

- All Classes

- Summary:
- Nested |
- Field |
- Constr |
- Method

- Detail:
- Field |
- Constr |
- Method
